# Simple Sequence Repeats Provide a Substrate for Phenotypic Variation in the *Neurospora crassa* Circadian Clock

**DOI:** 10.1371/journal.pone.0000795

**Published:** 2007-08-29

**Authors:** Todd P. Michael, Sohyun Park, Tae-Sung Kim, Jim Booth, Amanda Byer, Qi Sun, Joanne Chory, Kwangwon Lee

**Affiliations:** 1 Department of Plant Pathology, Cornell University, Ithaca, New York, United States of America; 2 Plant Biology Laboratory, The Salk Institute, La Jolla, California, United States of America; 3 Department of Biological Statistics and Computational Biology, Cornell University, Ithaca, New York, United States of America; 4 Cornell Theory Center, Cornell University, Ithaca, New York, United States of America; 5 Howard Hughes Medical Institute, The Salk Institute for Biological Studies, La Jolla, California, United States of America; University of British Columbia, Canada

## Abstract

**Background:**

WHITE COLLAR-1 (WC-1) mediates interactions between the circadian clock and the environment by acting as both a core clock component and as a blue light photoreceptor in *Neurospora crassa*. Loss of the amino-terminal polyglutamine (NpolyQ) domain in WC-1 results in an arrhythmic circadian clock; this data is consistent with this simple sequence repeat (SSR) being essential for clock function.

**Methodology/Principal Findings:**

Since SSRs are often polymorphic in length across natural populations, we reasoned that investigating natural variation of the WC-1 NpolyQ may provide insight into its role in the circadian clock. We observed significant phenotypic variation in the period, phase and temperature compensation of circadian regulated asexual conidiation across 143 *N. crassa* accessions. In addition to the NpolyQ, we identified two other simple sequence repeats in WC-1. The sizes of all three WC-1 SSRs correlated with polymorphisms in other clock genes, latitude and circadian period length. Furthermore, in a cross between two *N. crassa* accessions, the WC-1 NpolyQ co-segregated with period length.

**Conclusions/Significance:**

Natural variation of the WC-1 NpolyQ suggests a mechanism by which period length can be varied and selected for by the local environment that does not deleteriously affect WC-1 activity. Understanding natural variation in the *N. crassa* circadian clock will facilitate an understanding of how fungi exploit their environments.

## Introduction

Circadian clocks are internal timing mechanisms found across all kingdoms that synchronize the biological process of an organism with its local environment [1]–[3]. The period, or duration of each circadian cycle is about 24 hours. However, daily environmental cues such as light/dark and temperature cycles can set, or entrain, the period to the exact 24 hour period of the day. Entrainment to the ambient 24 hour period partitions biological processes to the correct time of day, or phase, in relation to environmental cues. Furthermore, it is important for the circadian clock to maintain a constant period over a range of physiological temperatures; hence, the clock is temperature compensated and its temperature coefficient (Q_10_) is lower than normal enzymatic reactions [4]. While the overall mechanism of the circadian clock is conserved across all kingdoms, the molecular details vary by organism.

The haploid filamentous fungus *Neurospora crassa* serves as an important model system for dissection of the molecular mechanisms of the circadian clock [1]. Four key clock components discussed in this study are WHITE COLLAR-1 (WC-1), WHITE COLLAR-2 (WC-2), FREQUENCY (FRQ), and VIVID (VVD). WC-1 and WC-2 are GATA-type zinc-finger (ZnFn) transcription factors that interact via their PER-ARNT-SIM (PAS) domains [5], to form the WHITE COLLAR COMPLEX (WCC). The WCC induces *frq* transcript levels through direct binding to the *frq* promoter [6]. VVD, another PAS protein, plays key roles in resetting the rhythm at dusk by promoting degradation of *frq* RNA, and in preventing light resetting at dawn [7]. WC-1 activity can be partitioned by its protein domains into both dark (circadian clock) functions as well as light (blue-light sensing) functions [8]. A specialized flavin adenine dinucleotide-binding PAS domain, LOV domain, is required for blue light sensing [6], [9], [10], whereas the ZnFn, nuclear localization signal and amino-terminal polyglutamine (NpolyQ) domain are required for clock function in continuous dark conditions [8], [11], [12]. Consistent with the NpolyQ having a clock specific function, two *wc-1* alleles lacking the NpolyQ, *wc-1^MK1^* and *wc-1^rhy-2^*, loose WC-1 mediated clock function in continuous dark conditions yet retain light responsiveness. While the exact mechanism of NpolyQ function remains unknown, the NpolyQ may serve as the activation domain required for *frq* transcription upon WCC binding [13], [14].

Underlying the WC-1 NpolyQ is a simple sequence repeat (SSR), which is a site of natural variation within populations due to slippage of DNA polymerase [15], [16]. For example, the circadian clock protein PERIOD in both *Drosophila melanogaster* and *Homo sapiens* harbours SSRs that are associated with known clock phenotypes across natural populations [17], [18]. There is variation in the circadian clock across natural populations of many eukaryotes [17]–[22]. Variation in the circadian clock reflects the environment of an organism and confers fitness by matching internal period with that of local daylength [17], [19], [23]. We hypothesized that the NpolyQ may be a site of variation across *N. crassa* populations thereby providing a mechanistic link between the environment and the circadian clock.

*N. crassa* accession collections are isolated from recently burned sub-tropical landscapes and, more recently, from temperate environments [24], [25]. While *N. crassa* accessions have been instrumental in physiological, ecological and inheritance studies [24]–[27], they have yet to be exploited for circadian biology due to technical issues associated with measuring clock regulated asexual reproduction (conidiation). Circadian regulated conidiation (banding) serves as an accurate phenotypic readout of the endogenous cellular clock and is measured with the race tube assay [1]. However, the race tube assay requires a genetic mutation band (*bd*) to mitigate the adverse effects of CO_2_ accumulation [28]. We developed the ‘Inverted Race Tube Assay’ to directly measure the clock parameters in *N. crassa* accession lacking the *bd* mutation [29]. In this study we describe extensive circadian phenotypic and clock genotypic variation across 143 *N. crassa* accessions, and we link variation at the WC-1 NpolyQ to period length in these populations.

## Results

### Phenotypic variation in the circadian clock across 143 accessions

**Figure 1:**
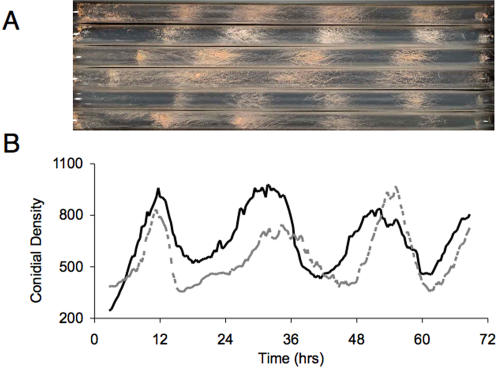
Phenotypic variation in pigmentation, period and phase in *N. crassa* conidiation revealed by inverted race tube assay. Representative accessions are shown to highlight variation in pigmentation (A), period and phase (B). The accessions in A are, from top to bottom, FGSC#4833, FGSC#852, FGSC#4829, FGSC#5914, FGSC#4817 and FGSC#4723. Conidial density in B was estimated using the Chrono program and plotted as a line graph for visual effect. The accessions in B are FGSC#6634 (black) and FGSC#3968 (grey). Accessions were grown at 25°C and represent one replicate to highlight circadian regulated conidiation (banding).

To gain more insight into natural variation in the *N. crassa* circadian clock, we obtained 143 randomly chosen *N. crassa* accessions from the ‘Perkins Collection’ at the Fungal Genetics Stock Center that were collected from around the world (43° North to 23° South, and from North America to Asia; Table S1) [26]. While the accessions represent diverse longitudes and latitudes, there is a bias towards the sub-tropics (Table S1). We measured circadian regulated asexual conidiation (banding) as a reliable phenotypic readout of the endogenous cellular clock [29]. The 143 accessions were assayed in continuous dark for five days at two constant temperatures, 20°C and 25°C. Utilizing the banding phenotype, we measured the circadian parameters of period as the duration between daily conidiation fronts (bands), of phase as the timing of the first band after release into continuous conditions, and of temperature compensation as the difference between the period of banding at 20°C and 25°C (see Materials and Methods for more details) [29]. We also recorded variation in both pigmentation (Figure 1A) and the daily timing of banding across the accessions (Figure 1B).

**Figure 2:**
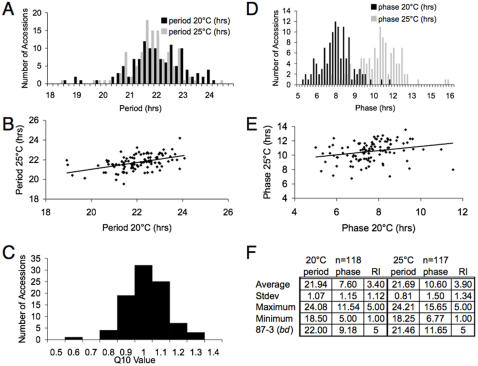
Significant phenotypic variation in period, phase and temperature compensation across 143 *N. crassa accessions.* Circadian period (A, B) and phase (D, E) were estimated using the Chrono program for 118 and 117 accessions at 20°C (grey) and 25°C (black) respectively that had an average rhythmicity index (RI) ≥ 3 (Materials and Methods). Period (B), temperature compensation (C) and phase (E) were analyzed for the 106 accessions with a RI≥3 at both temperatures. Temperature compensation (C) was measured as Q10 (Materials and Methods). (F) Summary of period, phase and RI across the rhythmic accessions at 20°C and 25°C. 87-3 is the *N. crassa* reference accession that harbours the *bd* mutation and is the primary lab strain. All values presented are an average of four biological replicates (Table S1).

Not all accessions displayed a scorable banding phenotype under the two temperatures tested. However, after visual inspection of banding for all of the accessions, we noted five distinct banding classes based on conidial density. In order to quantitatively score the accessions for banding across the circadian experiments, we designated each banding class with a banding score, or rhythmicity index (RI), between 1 and 5 with 1 representing no banding and 5 representing clear banding (see Materials and Methods). Since the RI was designed to be a quantitative measure of banding and conidial density, we averaged across replicates to determine which accessions to retain for analysis of circadian parameters. The average RI across the accessions was 3.4 and 3.9 at 20°C and 25°C, respectively (Figure 2A, F). At 20°C and 25°C 118 and 117 of the 143 accessions, respectively, displayed scorable banding which we defined as a RI≥3. We retained these accessions for further analysis.

The average periods of the accessions at 20°C and 25°C in continuous dark conditions were about 2 hours shorter than the natural 24 hour period of the day (21.94 and 21.69 hours, respectively). This average period was similar to that of the 87-3 reference strain that harbors the *bd* mutation (Figure 2F). While the ranges of periods for the accessions (18 to 24 hours) at 20°C and 25°C were similar (Figure 2A), the circadian period of each accession responded differently to temperature (Figure 2B). Since the circadian clock is temperature compensated, the period of the clock should not change significantly under different physiological temperatures. The Q_10_ value (temperature coefficient) describes the change in the rate of a biological process when the temperature is increased by 10°C, and has been used to quantitatively describe the effect of temperature on the circadian clock [4]. Therefore, we determined the Q_10_ value for each accession (Materials and Methods), and found variation in temperature compensation across the accessions (Figure 2C). We also found variation in the phase of the conidial banding on the first day of constant darkness across the population of accessions (Figure 2D and 2F). In contrast to period length, the average phase varied between the temperatures tested (7.6 and 10.76 hours; Figure 2D, E). In summary, we observed phenotypic variation in circadian period, phase and temperature compensation across the 143 *N. crassa* accessions.

### Genetic variation in clock genes across 143 *N. crassa* accessions

**Figure 3:**
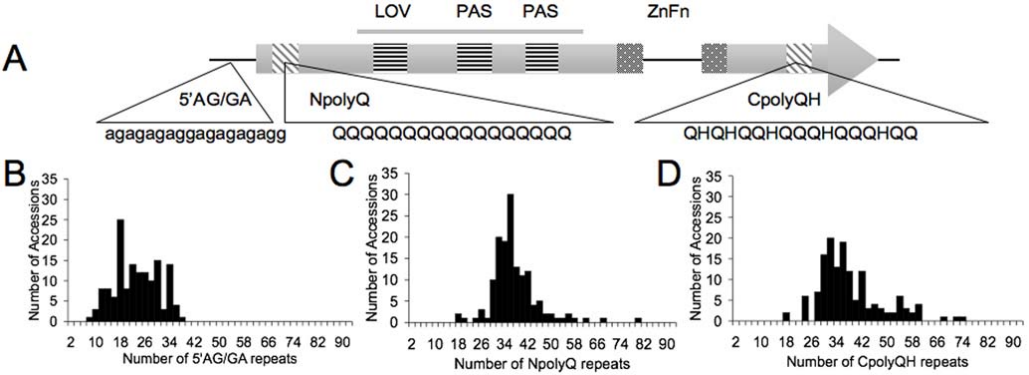
Extensive genotypic variation in WC-1 SSR. (A) The *wc-1* gene contains a 5′UTR, an intron, a 3′UTR (solid line) and two exons (boxes). WC-1 protein structure: AD, activation domain (striped boxes); LOV, light, oxygen and voltage domain (horizontal line boxes); PAS, PER-ARNT-SIM domain (horizontal line boxes); ZnFn, GATA-type zinc transcription factor DNA binding domain (grey dotted boxes). The number WC-1 5′AG/GA (B), NpolyQ (C) and CpolyQH (D) repeats were estimated across 143 *N. crassa* accessions (Materials and Methods). Repeat number was confirmed by sequencing the repeats in multiple accessions (Figure S1 and Table S1).

In order to link circadian phenotypic variation with molecular mechanisms, we explored the genetic variation of well-characterized *N. crassa* clock genes across the 143 accessions. Our primary interest was in the WC-1 gene as it encodes an activation domain (NpolyQ) that is necessary for clock function and contains an SSR [11], [12], [30]. Upon inspection of the predicted WC-1 ORF we noted two additional SSRs: an AG/GA repeat in the 5′ untranslated region (5′AG/GA) and a polyglutamine/histidine repeat in the carboxyl-terminal region (CpolyQH) (Figure 3A and Figure S1). To test the hypothesis that the molecular variation in WC-1 plays a role in phenotypic variation, we surveyed the size variation of SSRs in WC-1 across the accessions (Figure 3A). In order to assess the size variation in these three SSRs, we designed primers that flank the repeat regions and scored the size of the repeat regions across the 143 accessions. We also verified the length of the SSRs by sequencing accessions with different SRR lengths (Figure S1; Materials and Methods). While the 5′AG/GA, NpolyQ and CpolyQH SSRs displayed continuous variation across the accessions, the average number of repeats (22, 36 and 37, respectively) was similar to the *N. crassa* reference strain 87-3 (27, 39, and 35, respectively) (Figure 3B–D and Table S1).

**Figure 4:**
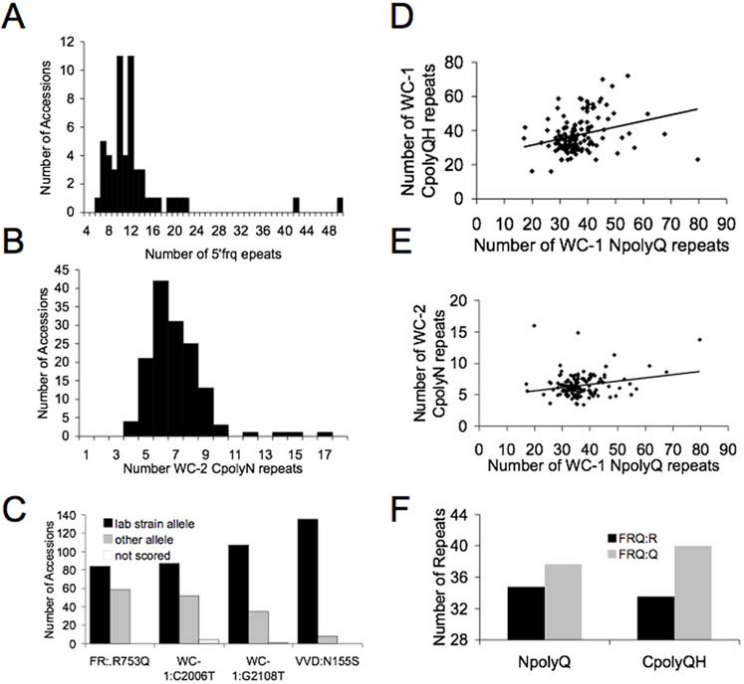
Genotypic variation WC-1, WC-2, FRQ and VVD. The number of 5′frq (A) and WC-2 CpolyN (B) repeats were estimated in the *N. crassa* accessions. (C) *wc-1*, *frq* and *vvd* SNPs were scored across 143 accessions. The allele frequency of the reference strain (black) is shown in comparison to the other allele (grey) across the accessions. (D–E) Associations between repeat length of NpolyQ and CpolyQH (D), and WC-2 CpolyN and NpolyQ (E). (F) The average length of both the NpolyQ and CpolyQH were significantly different by a student's t-test when grouped for the presence of the FRQ: R753 or FRQ: Q753 allele (p<0.05). All data is available in Table S1.

Due to sequence variation in the WC-1 SSRs, we asked if SSR polymorphisms are a common feature of other *N. crassa* circadian clock components. We evaluated the gene models of the clock genes, WC-2, FREQUENCY (FRQ), and VIVID (VVD), to determine if they contain potential SSRs. While we found a tri-nucleotide repeat in the *frq* promoter (5′frq) that varied between 5 and 50 ACC repeats (Figure 4A) we were only able to reliably score fifty-four accessions. Additionally, we found a carboxyl-terminal polyasperigine repeat (CpolyN) in WC-2 that varied between 3 and 16 repeats (Figure 4B).

We also sequenced *wc-1*, *wc-2*, *vvd* and *frq* in select accessions with either a large or small number of SSRs in order to identify potential single nucleotide polymorphisms (SNP). For *wc-1* we sequenced a 1454 base pair region surrounding the three PAS/LOV domains in 12 accessions and found a total of 40 nucleotide differences. These nucleotide polymorphisms did not change any of the amino acids in the PAS/LOV domains but did change four amino acids outside the PAS/LOV domains (Figure S2). Tajima's D test (−0.3952) for the PAS/LOV domains could not reject the null hypothesis of neutral mutation [31]. The lack of variation in the PAS/LOV domains was in stark contrast to the amount of SSR variation in the WC-1 protein. This result was consistent with the importance of the PAS/LOV domain in WC-1 mediated circadian and light signalling functions. We sequenced the entire *wc-2* gene in two accessions (FGSC#3974 and FGSC#4832) and identified 26 nucleotide changes and 5 amino acid changes and an additional amino-terminal twelve base pair (four amino acid) SSR in the coding region (Figure S3). We sequenced the entire *vvd* gene in eight accessions and identified 24 nucleotide changes and six amino acid changes in the coding region (Figure S4). In contrast to WC-1, we found amino acid changes in the PAS domains of both WC-2 and VVD. Finally we sequenced *frq* in the accession with the largest WC-1 NpolyQ repeat (FGSC#3974). We found 26 nucleotide changes and 17 amino acid changes in *frq* and FRQ between 87-3 and FGSC#3974 (Figure S5). We developed markers to score a number of the SNPs across the 143 accessions (Materials and Methods) and found multiple SNPs that were shared across the population (Figure 4C).

The common mechanism of the circadian clock relates WC-1, WC-2 and FRQ to one another at the molecular and genetic level [1]. Since the central parameters of the circadian clock (period, phase and temperature compensation) depend on the relationship between the activity of WC-1, WC-2 and FRQ, we hypothesized that the variation in these clock loci may be genetically associated (correlated) across the accessions. Consistent with WC-1 SSRs experiencing similar expansion pressure, we found significant correlations between all three WC-1 SSRs (Figure 4D, Table 1). The WC-2 CpolyN was significantly correlated with the WC-1 5′AG/GA but not with the CpolyQH and NpolyQ (Table 1). Both the WC-1 NpolyQ and CpolyQH were correlated with FRQ:R753Q (p = 0.045 and p<0.001 respectively, based on the two-sample t-test [28]), and in both cases the FRQ:Q allele was correlated with a higher repeat number in WC-1 (Figure 4F). The genomic correlations between FRQ and WC-1 suggest that some selective pressure, possibly the need to maintain circadian period within certain limits, maintains the genetic relationship between these two loci. Environmental pressures on the period of the circadian clock may be driving FRQ and WC-1 into linkage disequilibrium. However, we did not test for linkage disequilibrium in this study. In summary, we identified significant variation in the circadian clock genes WC-1, WC-2, FRQ and VVD. For WC-1 and WC-2, SSRs provided the majority of the variation at the protein level and this variation was associated with variation in FRQ.

**Table 1:** Associations between genotype, phenotype and circadian parameters across *Neurospora crassa* accessions at 25°C

	Latitude*		Period		Phase		WC1 5′AGA		WC1 NpolyQ		WC1 CpolyQH		WC-2 CpolyN	
	r	p	r	p	r	p	r	p	r	p	r	p	r	p
Period	**0.21** *	**0.022**												
Phase	**0.28** *	**0.002**	0.12	0.240										
WC-1 5′AGA	0.03*	0.708	**0.23**	**0.020**	0.13	0.176								
WC-1 NpolyQ	**0.20** *	**0.030**	**0.26**	**0.008**	0.10	0.303	**0.32**	**0.001**						
WC-1 CpolyQH	**0.24** *	**0.010**	**0.21**	**0.036**	0.00	0.971	**0.63**	**0.000**	**0.39**	**0.000**				
WC-2 CpolyN	**0.31** *	**0.001**	0.60	0.575	0.00	0.990	**0.27**	**0.006**	0.02	0.865	0.16	0.095		
FRQ:R753Q	**0.48** *	**0.000**	0.00	0.803	**0.38**	**0.000**	0.15	0.124	0.19	0.059	**0.29**	**0.003**	0.03	0.669
VVD:N155S	0.01*	0.264	0.04	0.633	0.03	0.672	0.06	0.511	0.14	0.167	0.10	0.301	**0.25**	**0.010**
WC-1:C2006T	0.14*	0.327	0.16	0.271	0.05	0.860	0.20	0.126	0.30	0.009	**0.38**	**0.000**	0.03	0.955
WC-1:G2108T	**0.33** *	**0.001**	0.18	0.203	**0.30**	**0.008**	**0.39**	**0.000**	**0.29**	**0.014**	0.20	0.127	0.09	0.637

* Accessions RI≥3.

Unmarked: 104/143 accessions used for this analysis; accessions were rhythmic under both temperatures and RI≥3

Linear modeling implemented in R; bold highlight signifies significance p<0.05

### Circadian genotypes and phenotypes are associated with latitude of collection

To test if the underlying genotypic variation accounts for the observed phenotypic variation, we asked if the length of the WC-1 NpolyQ is responsible for variation in the period (Table 1). We found that the WC-1 SSRs: 5′AG/GA, NpolyQ and CpolyQH, all correlated with the period (r∼0.25, p<0.05). Additionally, both the WC-1 NpolyQ and CpolyQH correlated with latitude of collection (r∼0.26, p<0.01). Finally, both period and phase were correlated with latitude of collection (Table 1). In the flowering plant model *Arabidopsis thaliana*, period increases with latitude [19]; however, in our *N. crassa* populations we observed the opposite relationship as accessions with longer periods reside near the equator.

To investigate possible multivariate relationships between NpolyQ, latitude and FRQ: R753Q, and their combined effect on period, we conducted a multiple linear regression analysis with period as the response variable. All forms of two-factor interactions, as well as three-factor interactions between the three predictors were included in the model. The parameter estimates and associated tests are provided in Table 2. The model fit revealed a significant interaction between NpolyQ and latitude in their effect on period (p = 0.020). In particular, there was generally a negative (linear) association between period length and NpolyQ length and this negative association strengthened with increasing latitude. No other interaction effects were found to be statistically significant. These results suggested that increased NpolyQ length conferred longer circadian periods to accessions closer to the equator.

**Table 2:** Parameter estimates and tests from a multiple linear regression of period length on latitude, *wc-1* and *frq* genotypes; NpolyQ length and FRQ: R753Q, respectively.

Parameter	Estimate	Std. Error	T-statistic	P-value
Intercept	21.4706	0.9348	22.968	<.0001
Latitude	0.1592	0.0758	2.101	0.0380
NpolyQ	0.0161	0.0252	0.639	0.5243
R753Q R	0.9230	1.4907	0.619	0.5371
Lat:NpolyQ	−0.0052	0.0022	−2.369	0.0196
Lat:R753Q	−0.1292	0.0915	−1.412	0.1608
NpolyQ:R753Q	−0.0242	0.0403	−0.601	0.5489
Lat:NpolyQ:R753Q	0.0036	0.0026	1.403	0.1635

### WC-1 NpolyQ length segregates with circadian period

**Figure 5:**
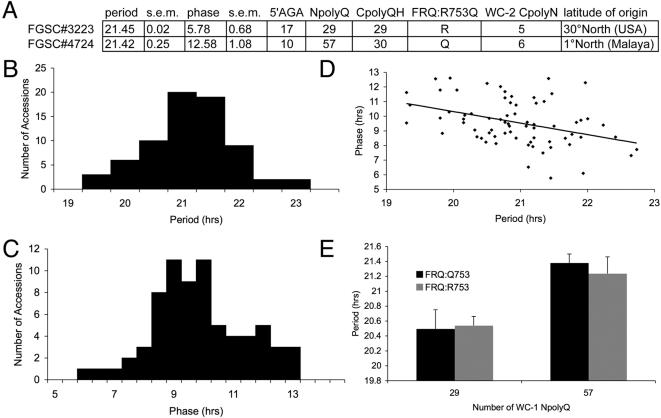
WC-1 genotype segregates with period length in a cross between two *N. crassa* accessions. (A) Period, phase, genotype and latitude of collection information for the parental strains FGSC#3223 and FGSC#4724 at 25°C. Period (B) and phase (C) were measured for both parental strains (FGSC#3223 and FGSC#4274) as well as for 69 progeny from the cross (Table S2). (D) Association between period and phase of the progeny. (E) Period length was significantly associated with WC-1 genotype, regardless of FRQ genotype (*P*<0.001). Since FRQ and WC-1 are on the same chromosome (linkage group), we scored FRQ: Q753R for crossover events between the two loci. Eleven crossover events were identified in the 69 progeny, FRQ: Q753 (black) and FRQ: R753 (grey). Average period and phase of two biological replicates are presented, and data available in Table S2.

We wanted to test if the polymorphism in WC-1 NpolyQ contributes to variation in clock phenotypes. Therefore, we crossed two accessions that have different NpolyQ lengths yet share similar CpolyQH lengths (Figure 5A). We used the inverted race tube assay to score the period and phase of progeny from the cross (Figure 5, Table S2). Consistent with transgressive segregation, the period of the progeny was outside the range observed for either parental line (Figure 5B, C). We found an association between period and phase as progeny with longer period had earlier phases (Figure 5D). We genotyped the cross for the WC-1 NpolyQ and found that period length co-segregated with NpolyQ length (Figure 5E, p<0.001). Since FRQ and WC-1 are located on the same chromosome, we also genotyped FRQ:R753Q to determine if FRQ was responsible for the differences in period . We observed a direct relationship between NpolyQ and period length that was independent of the FRQ genotype as the progeny with longer NpolyQs (Q = 57) had significantly longer periods than those progeny with the shorter NpolyQ (Q = 29), regardless of the genotype at the FRQ locus (Figure 5E). In contrast to natural populations where WC-1 and FRQ genotypes are associated, the progeny of this cross allowed us to separate the contributions of WC-1 and FRQ on the period. We conclude that the genetic link between WC-1 and FRQ was not required for the period effects and that the region surrounding WC-1 was most likely responsible for the effects on period. The WC-1 NpolyQ conferred a longer period and this is consistent with our observation that latitude-dependent period length also increased with WC-1 NpolyQ length. Taken together, these results argue that the WC-1 NpolyQ provides a genetic mechanism for *N. crassa* accessions to adapt to their local environment (latitude).

## Discussion

In this study we described natural phenotypic and genotypic variation in the *N. crassa* circadian clock. We established that the natural period length of the *N. crassa* circadian clock is 22 hours, two hours shorter than the external period of daily light and temperature cycles. We also linked variation at the core circadian clock and light signalling locus, WC-1, with the period of circadian regulated asexual conidiation. In natural populations of *N. crassa* WC-1 and FRQ genotypes are genetically linked and control the period. However, we genetically separated the WC-1 and FRQ alleles and demonstrated that the SSR polymorphism in WC-1 accounted for the period phenotype (Figure 5). We provided evidence that variation in the circadian clock was associated with latitude of collection, which suggested that the WC-1 genotype provided an adaptive advantage in natural populations. The data presented here support a functional role for the WC-1 NployQ in the *N. crassa* circadian clock and implicated SSRs as a source of variation in the *N. crassa* circadian system. Since WC-1 played a dual role as a core circadian clock component and as a photoreceptor, moderate changes in its activity through SSR length variation would allow fine-tuning of WC-1 activity while maintaining overall clock functionality. SSRs may provide the necessary variation for *N. crassa* accessions to colonize new environments or to better exploit their current environment.

The ecological significance of asexual conidiation in *N. crassa* is poorly understood, despite the fact that its genetic regulation is well characterized [32]–[34]. Conidiation and aerial dispersion are key features of terrestrial fungi [35]; furthermore, what is known of their ecological significance emerges from research on pathological fungi [36]–[38]. *Magnaporthe oryzae*, a pathogenic fungus, releases its spores late at night and into the early morning [39]. Similarly, under light/dark cycles *N. crassa* starts to conidiate during the dark period with maximal conidiation coinciding with dawn [40]. There are several potential advantages to dawn specific conidiation. Recent results link cell cycle with the circadian clock in *N. crassa*, which suggests that night-specific conidiation protects DNA from damaging UV rays [41]. Additionally, early morning warming of the air by the sun provides a dynamic dispersal mechanism [42] and early morning dew formation may protect spores from desiccation. The *N. crassa* accessions described in this study will be useful in further characterizations of the ecological significance of circadian regulated asexual reproduction in fungi.

The period length of conidiation in *N. crassa* accessions varied between 18 and 24 hours; this data was consistent with the amount of variation in natural populations of other species [17]–[22]. If most organisms experience a twenty-four hour day, why is there a range in period lengths within natural populations? In natural populations of *Drosophila* and *Arabidopsis* the period length is correlated with the latitude of collection [17], [19], supporting of idea that the clock increases an organism's fitness by matching the internal period with the external period of the environment to ensure that activities are phased to the correct time of day [23], [43]. While most organisms on Earth experience the same day length (24 hours), daylength (photoperiod and thermoperiod) varies by latitude and season. Therefore the circadian clock must integrate daylength information with biological activities so that biological activities will be phased to the correct time of day [21]. The primary function of the circadian clock is to phase the biological activities to the correct time of day. For instance, in *Arabidopsis* the circadian clock controls flowering time by ensuring that specific genes are expressed at the correct phase of the day, in a season specific fashion [44]. Rather than modulating the phase directly, *Arabidopsis* accessions from higher latitudes rely on longer periods to ensure proper phasing of biological activities under the longer photoperiods of the growing season [19].

In this study we detected a significant association between period, WC-1 NpolyQ and latitude of collection in *N. crassa* that is consistent with WC-1 NpolyQ dependent increases in period length in accessions that reside closer to the equator. While it would be speculation at this point to explain why period length increased closer to the equator, extrapolation from other systems suggests that it may play a role in ensuring the dawn specific release of spores at the correct time of year. This is an exciting hypothesis to test, and the data will provide insight into both fungal ecology as well as the adaptive significance of the circadian clock.

Why was the average period of the *N. crassa* circadian clock less than 24 hours? *Drosophila* populations have a similar average period length shorter than 24 hours [17] while the average period of *Arabidopsis* populations is longer than 24 hours [19], [20]. Theoretical modelling predicts periods less that 24 hours seen in *N. crassa* and *Drosophila* result in more robust oscillations in clock proteins and ensure stable entrainment to the environment [45]. This model is based on the rapid increase in *frq* transcript by light; however, recent research suggests that VVD prevents the clock from being reset at dawn (the dark to light transition) and that VVD promotes clock resetting at dusk (light to dark transition) by influencing the rate of *frq* transcript turnover [7]. VVD directly interferes with WCC [46], which suggests that dusk specific resetting of the clock reflects the activity of WC-1 at the *frq* promoter. Although the WC-1 NpolyQ is predicted to be an activation domain, [13], [14], the polyglutamine repeats also play a role in protein degradation [47]. Therefore, the WC-1 NployQ may mediate the specific kinetics of VVD mediated clock resetting at dusk to generate the two hour phase delay that is required to match it to the environment's 24 hour period.

SSRs provide an ideal mechanism for generating environment specific variation. In the mushroom *Coprinus cinereus*, mutants lacking the carboxy-terminal SSR of a WC-1/VVD ortholog *dst1* cannot sense light [48]. Since WC-1 plays a dual role as both a photoreceptor and as a core clock component, there are specific constraints on changes in its activity. For example, traditional mutagenesis has only identified arrhythmic *wc-1* alleles [49], which suggests that even minor changes in WC-1 lead to severe loss of functionality. Consistent with this data, we detected a low level of variation in the PAS/LOV region of WC-1 that is in contrast to the extensive variation present in the SSRs. Natural variation provides an array of *wc-1* alleles, which allows WC-1 to satisfy the general principle that core clock components will have short, long and arrhythmic alleles [50], [51]. Additionally, naturally occurring alleles define a role for NpolyQ in the circadian system and this may be a method of fine-tuning the circadian system to specific environmental conditions. Two different length WC-1 proteins are expressed from distinct promoters, one of which is a polypeptide lacking the NpolyQ domain [52]. This sophisticated regulation of WC-1 supports our view that the WC-1 NpolyQ plays a role in fine-tuning circadian system. Understanding how the environment acts on SSRs will provide new insight into the adaptive significance of the circadian clock.

## Materials and Methods

### *N. crassa* growing conditions and circadian race tube analysis

*N. crassa* accessions were obtained from the Fungal Genetics Stock Center (University of Missouri, Kansas City) and handled as described [11]. Circadian race tube experiments were performed with the Inverted Race Tube Assay in accessions that lack the *bd* mutation [29]. Race tubes were incubated in constant light for 24 hr and then moved to constant dark. To reduce the temperature effects of marking the growing front, only on the last day of the experiment was the growing front marked at the light to dark transfer. Not all of the 143 *N. crassa* accessions displayed scorable conidiation (banding). In order to quantify banding for use in further analysis, we developed a rhythmicity index (RI). Banding was scored on an RI scale from 1 to 5 with 1 representing no detectable banding, 2 representing only the first banding peak after transfer into darkness is detected, 3 representing rhythmic banding (first peak is pronounced while subsequent have a low density measure), 4 representing rhythmic banding (all peaks have equal density), 5 representing rhythmic banding with high density banding. Since the RI was a quantitative measure of both banding density and rhythmicity, we averaged these values across four replicates and analyzed the circadian parameters, via Chrono software package, of accessions whose average RI was greater than or equal to 3 [53]. Of the 143 accessions, 118 and 117 accessions had average RI≥3 at 20°C and 25°C, respectively. In our study phase was the estimated circadian time (regression analysis by Chrono program) of the first conidiation band after release into continuous dark. Period and phase data was expressed as the average and standard error of the mean (SEM) of at least four biological replicates. Q_10_ was calculated based on the formula,
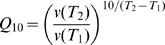
where, T2 = period at 25 C and T1 = period at 20 C [4].

Period and phase data for the cross between *N. crassa* accessions are the average and SEM of 2 biological replicates. Latitude of origin was estimated based on the reported city and country of collection provided by the Fungal Genetics Stock Center [26].

### Statistical Methods

#### Pearson Correlation Coefficient

We used the sample Pearson correlation coefficient to test for a linear association between two quantitative variables, *X* and *Y*. Specifically, suppose that (*x_i_, y_i_* ), *i = *1,…,*n*, are the measured values of *X* and *Y* for *n* accessions, then the sample Pearson correlation is given by
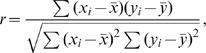
where *x̅* and *y̅* are the sample means of the two variables. The standard test for non-zero correlation assumes the data are bivariate normal, in which case an estimate of the standard error of *r* is given by
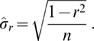
The t-statistic for testing the hypothesis of no correlation between *X* and *Y* is *T* = *r*/σˆ*_r_*. The p-value for the test is determined by comparing the observed *T* statistic to a t-distribution with *n*-2 degrees-of-freedom. We used the “cor.test” function in the R statistical package to perform these calculations.

#### Two-sample *T*-test

To compare two groups with respect to a quantitative response, *Y*, we used the two-sample *T*-statistic,
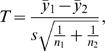
where *n_i_* and *y̅* *_i_* are respectively the sample size and sample mean for group *i, i = *1,2, and *s^2^* is the pooled sample variance,
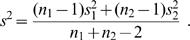
The p-value for assessing the hypothesis of no difference between the group means is determined by comparing the observed *T*-statistic to a t-distribution with *n*_1_+*n_2_*−2 degrees-of-freedom. We used the “t-test” function in the R statistical package to perform these calculations.

#### Multiple Linear Regression

To test for multivariate relationships in which the effects of several potential predictors on a quantitative response, *Y*, are assessed simultaneously, we used multiple linear regression analysis. Specifically, in this paper we investigated the joint effects of N = NpolyQ, L = latitude and the binary variable F = FRQ:R753Q on Y = period. We considered the multiple linear regression model with all possible interactions,
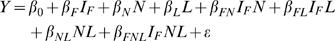
where *I_F_* is a zero-one indicator for the FRQ:Q/R alleles, the *β*′s are coefficients associated with the terms in the model, and *ε* is an error term to account for unexplained variation. We fit the model to the data using the “lm” function in the R statistical package (http://www.r-project.org/). The statistical significance of each effect can be assessed using a t-statistic equal to the ratio of each estimated coefficient to its standard error.

### Genotyping

Simple sequence repeats were identified in the *N. crassa* genomic sequence version 3 (http://www.broad.mit.edu/annotation/fungi/neurospora/). Primers were designed using the primer3 program [54]. All simple sequence repeats were scored by amplification of a 50–200 bp product with primers that flank the repeats. PCR fragments were resolved on 3% agarose gels and their lengths were estimated using Quality One software (Biorad) against a 50 bp DNA ladder (Invitrogen). Based on sequencing data from a limited number of accessions we were able to estimate the size of an SRR within 1 bp. In order to score SNP, DNA was amplified using gene specific primers and amplification was verified via agarose gel electrophoresis. The PCR products were then digested with the appropriate enzyme that cut one allele but not the other, resolved on 3% agarose gels and were scored using the Quality One software (Biorad). All genotypes were entered into Microsoft Excel.

### Sequencing and alignments

We selected ‘extreme’ accessions for sequencing from based on WC-1 NpolyQ length; we defined extreme as being on one of the tails of the NpolyQ length distribution. *wc-1*, *wc-2*, *vvd* and *frq* genes were sequenced from genomic DNA as described [11]. DNA was amplified from the accessions (specified in the results) using gene specific primers, the amplification products were gel purified and Sanger sequenced. Sequence trace files were aligned and quality checked using SeqMan package in DNASTAR Lasergene 6 software suite. The sequence alignments and phylogenetic tree generation were carried out using ClustalW in MegAlign DNASTAR Lasergene 6 software suite with default settings. Sequence alignment figures were generated using Clustalx software using default settings.

## Supporting Information

Figure S1 Variation in WC-1 5′AG/GA, NpolyQ and CpolyQH. (A) Sequence variation in the WC-1 5′AG/GA repeat in accessions FGSC#4819, 2489, 4730, 6204, 4828, 4712, 4831, 4821, and 4721. (B) Sequence variation in the WC-1 NpolyQ repeat in accessions FGSC#2489, 3970, 3972, 6241, 4832, 2712, 4819, 3970A, and 3974. (C) Sequence variation in the WC-1 NpolyQ repeat in accessions FGSC#3974, 3972, 2712, 3199, 2227, 2489, 4706, 3359, and 3970. Choice of accessions, sequencing and alignment in Clustalx are described in Experimental procedures.
(2.50 MB TIF)

Figure S2 Variation in the wc-1 LOV/PAS/PAS region. Alignment of the region surrounding the three WC-1 PAS domains (LOV/PAS/PAS) of accessions FGSC#4712, 4713, 2227, 3972, 4706, 3199, 3970, 2489, 4730, 4825, 3974, 4722, and 3359. Choice of accessions, sequencing and alignment in Clustalx are described in Experimental procedures.
(2.50 MB TIF)

Figure S3 Variation in WC-2. Gene model for wc-2 including its domain coding structure: PAS, PAS domain; ZnF, zinc finger; and AD, activation domain. wc-2 has two SSR, the NpolyGX and CpolyN. WC-2 protein sequence from the sequence of two *N. crassa* accessions FGSC#3974 and 4832 and comparison to the 87-3 reference strain. Lines over protein sequence demarcate respective domains. Choice of accessions, sequencing and alignment in Clustalx are described in Experimental procedures.
(2.50 MB TIF)

Figure S4 Variation in VVD. VVD protein sequence deduced from the sequence of *N. crassa* accessions FGSC#2227, 6421, 87-3 (a reference strain), 4826, 4832, 3972, 3974, 3970, and 4819. Phylogenetic tree of VVD proteins across accessions was created using ClustalW alignment in MegAlign DNASTAR Lasergene 6 software suite with default settings. Choice of accessions, sequencing and alignment in Clustalx are described in Experimental procedures.
(2.50 MB TIF)

Figure S5 Variation in FRQ. FRQ protein sequence deduced from sequencing *N. crassa* accessions FGSC#3974 and sequenced lab strain 74a (FGSC#2489). Choice of accessions, sequencing and alignment in Clustalx are described in Experimental procedures.
(2.50 MB TIF)

Table S1 *N. crassa* genotypes, circadian phenotypes and latitudes of collection. *Rhythmicity Index; 1: arrhythmic, no rhythmic banding phenotype, 2: only the first peak after transferred to a constant condition but no clear peak after that peak, 3: rhythmic banding, first peak is pronounced while other peaks are weak, 4: rhythmic banding, no pronounced first peak, 5: robust rhythmic banding FGSC#: fungal genetics stock number; mat: mating type; lat: latitude; hem: hemisphere, N: North, S: South per: circadian period; ph: circadian phase on the first day %87-3 is a lab strain
(0.07 MB XLS)

Table S2 Circadian period and phase for progeny of the *N. crassa* cross FGSC#3223 X FGSC#4724
(0.03 MB XLS)

## References

[1] Dunlap JC (2006). Proteins in the Neurospora circadian clockworks.. J Biol Chem.

[2] Wijnen H, Young MW (2006). Interplay of circadian clocks and metabolic rhythms.. Annu Rev Genet.

[3] Hotta CT, Gardner MJ, Hubbard KE, Baek SJ, Dalchau N (2007). Modulation of environmental responses of plants by circadian clocks.. Plant Cell Environ.

[4] Ruoff P, Vinsjevik M, Rensing L (2000). Temperature compensation in biological oscillators: a challenge for joint experimental and theoretical analysis.. Comments Theor Biol.

[5] Gu YZ, Hogenesch JB, Bradfield CA (2000). The PAS superfamily: sensors of environmental and developmental signals.. Annual Review of Pharmacology&Toxicology.

[6] Froehlich AC, Liu Y, Loros JJ, Dunlap JC (2002). White Collar-1, a circadian blue light photoreceptor, binding to the *frequency* promoter.. Science.

[7] Elvin M, Loros JJ, Dunlap JC, Heintzen C (2005). The PAS/LOV protein VIVID supports a rapidly dampened daytime oscillator that facilitates entrainment of the Neurospora circadian clock.. Genes Dev.

[8] Cheng P, Yang Y, Wang L, He Q, Liu Y (2003). WHITE COLLAR-1, a multifunctional neurospora protein involved in the circadian feedback loops, light sensing, and transcription repression of wc-2.. J Biol Chem.

[9] Christie JM, Salomon M, Nozue K, Wada M, Briggs WR (1999). LOV (light, oxygen, or voltage) domains of the blue-light photoreceptor phototropin (nph1): Binding sites for the chromophore flavin mononucleotide.. Proc Natl Acad Sci USA.

[10] He Q, Cheng P, Yang Y, Wang L, Gardner KH (2002). White Collar-1, a DNA binding transcription factor and a light sensor.. Science.

[11] Lee K, Dunlap CJ, Loros JJ (2003). Roles for WHITE COLLAR-1 in Circadian and General Photoperception in *Neurospora crassa*.. Genetics.

[12] Toyota K, Onai K, Nakashima H (2002). A new *wc-1* mutant of *Neurospora crassa* shows unique light sensitivity in the circadian conidiation rhythm.. Mol Gen Genomics.

[13] Ballario P, Vittorioso P, Magrelli A, Talora C, Cabibbo A (1996). White collar-1, a central regulator of blue light responses in *Neurospora*, is a zinc finger protein.. EMBO J.

[14] Liu Y (2003). Molecular mechanisms of entrainment in the Neurospora circadian clock.. J Biol Rhythms.

[15] Borstnik B, Pumpernik D (2002). Tandem Repeats in Protein Coding Regions of Primate Genes.. Genome Res.

[16] Levinson G, Gutman G (1987). Slipped-strand mispairing: a major mechanism for DNA sequence evolution.. Mol Biol Evol.

[17] Sawyer LA, Hennessy JM, Peixoto AA, Rosato E, Parkinson H (1997). Natural Variation in a Drosophila Clock Gene and Temperature Compensation.. Science.

[18] Nadkarni NA, Weale ME, von Schantz M, Thomas MG (2005). Evolution of a Length Polymorphism in the Human PER3 Gene, a Component of the Circadian System.. J Biol Rhythms.

[19] Michael TP, Salome PA, Yu HJ, Spencer TR, Sharp EL (2003). Enhanced Fitness Conferred by Naturally Occurring Variation in the Circadian Clock.. Science.

[20] Edwards KD, Lynn JR, Gyula P, Nagy F, Millar AJ (2005). Natural allelic variation in the temperature-compensation mechanisms of the Arabidopsis thaliana circadian clock.. Genetics.

[21] Pittendrigh CS, Takamura T (1989). Latitudinal clines in the properties of a circadian pacemaker.. J Biol Rhythms.

[22] Brown SA, Fleury-Olela F, Nagoshi E, Hauser C, Juge C (2005). The period length of fibroblast circadian gene expression varies widely among human individuals.. PLoS Biol.

[23] Dodd AN, Salathia N, Hall A, Kevei E, Toth R (2005). Plant circadian clocks increase photosynthesis, growth, survival, and competitive advantage.. Science.

[24] Jacobson DJ, Powell AJ, Dettman JR, Saenz GS, Barton MM (2004). Neurospora in temperate forests of western North America.. Mycologia.

[25] Jacobson DJ, Dettman JR, Adams RI, Boesl C, Sultana S (2006). New findings of Neurospora in Europe and comparisons of diversity in temperate climates on continental scales.. Mycologia.

[26] Turner BC, Perkins DD, Fairfield A (2001). Neurospora from Natural Populations: A Global Study.. Fungal Genetics and Biology.

[27] Bhat A, Noubissi FK, Vyas M, Kasbekar DP (2003). Genetic Analysis of Wild-Isolated Neurospora crassa Strains Identified as Dominant Suppressors of Repeat-Induced Point Mutation.. Genetics.

[28] Sargent ML, Kaltenborn SH (1972). Effects of medium composition and carbon dioxide on circadian conidiation in Neurospora.. Plant Physiol.

[29] Park S, Lee K (2004). Inverted Race Tube Assay for Circadian Clock Studies of the Neurospora Accessions.. Fungal Gen Newsl.

[30] Karaoglu H, Lee CM, Meyer W (2005). Survey of simple sequence repeats in completed fungal genomes.. Mol Biol Evol.

[31] Tajima F (1989). Statistical method for testing the neutral mutation hypothesis by DNA polymorphism.. Genetics.

[32] Springer ML, Yanofsky C (1989). A morphological and genetic analysis of conidiophore development in Neurospora crassa.. Genes Dev.

[33] Springer ML (1993). Genetic control of fungal differentiation: the three sporulation pathways of Neurospora crassa.. Bioessays.

[34] Davis RH (2000). Neurospora, contributions of a model organism..

[35] James TY, Kauff F, Schoch CL, Matheny PB, Hofstetter V (2006). Reconstructing the early evolution of Fungi using a six-gene phylogeny.. Nature.

[36] Schmale DGI, Shields EJ, Bergstrom GC (2006). Night-time spore deposition of the fusarium head blight pathogen, Gibberella zeae, in rotational wheat fields.. Can J Plant Pathol.

[37] Brown JK, Hovmoller MS (2002). Aerial dispersal of pathogens on the global and continental scales and its impact on plant disease.. Science.

[38] Aylor DE, Taylor GS (1982). Aerial dispersal and drying of Peronospora tabacina conidia in tobacco shade tents.. Proc Natl Acad Sci U S A.

[39] Kim CK, Min HS, Yoshino R (1990). Epidemiological studies of rice blast disease caused by *Pyricularia oryzae* Cavara (III); Diurnal pattern of conidial release and dispersal under the natural conditions.. Ann Phytopath Soc Japan.

[40] Merrow M, Boesl C, Ricken J, Messerschmitt M, Goedel M (2006). Entrainment of the Neurospora circadian clock.. Chronobiol Int.

[41] Pregueiro AM, Liu Q, Baker CL, Dunlap JC, Loros JJ (2006). The Neurospora checkpoint kinase 2: a regulatory link between the circadian and cell cycles.. Science.

[42] Oke TR (1987). Boundary layer climates..

[43] Harmer SL, Hogenesch JB, Straume M, Chang H-S, Han B (2000). Orchestrated transcription of key pathways in *Arabidopsis* by the circadian clock.. Science.

[44] Yanovsky MJ, Kay SA (2002). Molecular basis of seasonal time measurement in Arabidopsis.. Nature.

[45] Kurosawa G, Goldbeter A (2006). Amplitude of circadian oscillations entrained by 24-h light-dark cycles.. J Theor Biol.

[46] Heintzen C, Loros JJ, Dunlap JC (2001). The PAS protein VIVID defines a clock-associated feedback loop that represses light input, modulates gating, and regulates clock resetting.. Cell.

[47] Sanchez I, Mahlke C, Yuan J (2003). Pivotal role of oligomerization in expanded polyglutamine neurodegenerative disorders.. Nature.

[48] Terashima K, Yuki K, Muraguchi H, Akiyama M, Kamada T (2005). The dst1 gene involved in mushroom photomorphogenesis of Coprinus cinereus encodes a putative photoreceptor for blue light.. Genetics.

[49] Dunlap JC, Loros JJ (2004). The Neurospora Circadian System.. J Biol Rhythms.

[50] Loros JJ, Dunlap JC (2001). Genetic and molecular analysis of circadian rhythms in *Neurospora*.. Annu Rev Physiol.

[51] Lakin-Thomas PL, Brody S (2004). CIRCADIAN RHYTHMS IN MICROORGANISMS: New Complexities.. Annual Review of Microbiology.

[52] Kaldi K, Gonzalez BH, Brunner M (2006). Transcriptional regulation of the Neurospora circadian clock gene wc-1 affects the phase of circadian output.. EMBO Rep.

[53] Roenneberg T, Taylor W (2000). Automated recordings of bioluminescence with special reference to the analysis of circadian rhythms.. Methods Enzymol.

[54] Rozen S, Skaletsky HJ (1998). Primer3.. http://www-genomewimitedu/genome_software/other/primer3html.

