# Supplementary figures and images for: Simple Sequence Repeats Provide a Substrate for Phenotypic Variation in the Neurospora crassa Circadian Clock
Source: PLoS One. 2007 Aug 29;2(8):e795. doi: 10.1371/journal.pone.0000795 (PMC1949147; doi:10.1371/journal.pone.0000795)

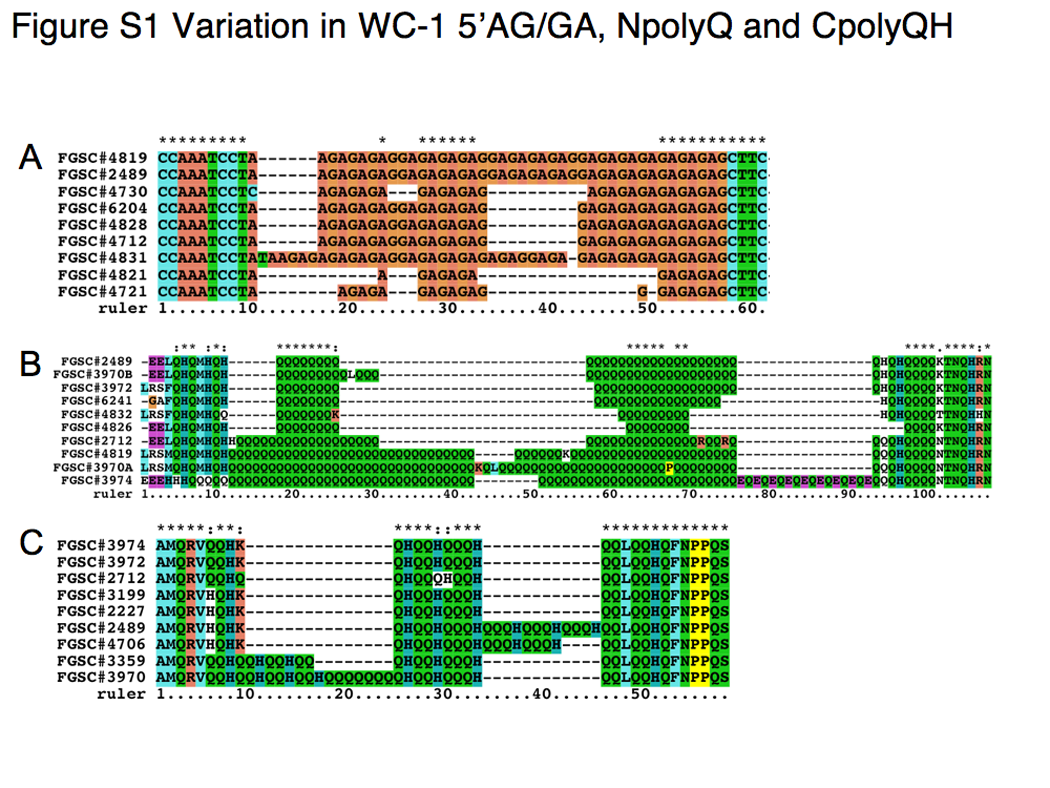

Supplement: Figure S1 — Variation in WC-1 5′AG/GA, NpolyQ and CpolyQH. (A) Sequence variation in the WC-1 5′AG/GA repeat in accessions FGSC#4819, 2489, 4730, 6204, 4828, 4712, 4831, 4821, and 4721. (B) Sequence variation in the WC-1 NpolyQ repeat in accessions FGSC#2489, 3970, 3972, 6241, 4832, 2712, 4819, 3970A, and 3974. (C) Sequence variation in the WC-1 NpolyQ repeat in accessions FGSC#3974, 3972, 2712, 3199, 2227, 2489, 4706, 3359, and 3970. Choice of accessions, sequencing and alignment in Clustalx are described in Experimental procedures. (2.50 MB TIF) [file pone.0000795.s001.tif]

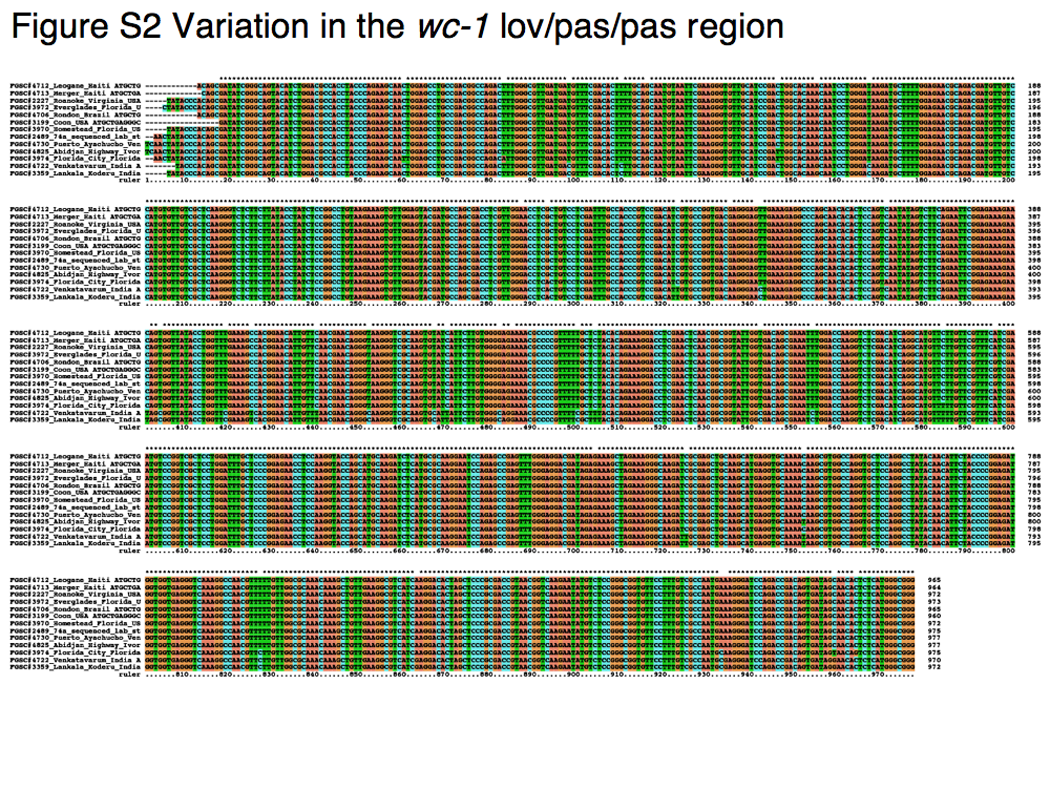

Supplement: Figure S2 — Variation in the wc-1 LOV/PAS/PAS region. Alignment of the region surrounding the three WC-1 PAS domains (LOV/PAS/PAS) of accessions FGSC#4712, 4713, 2227, 3972, 4706, 3199, 3970, 2489, 4730, 4825, 3974, 4722, and 3359. Choice of accessions, sequencing and alignment in Clustalx are described in Experimental procedures. (2.50 MB TIF) [file pone.0000795.s002.tif]

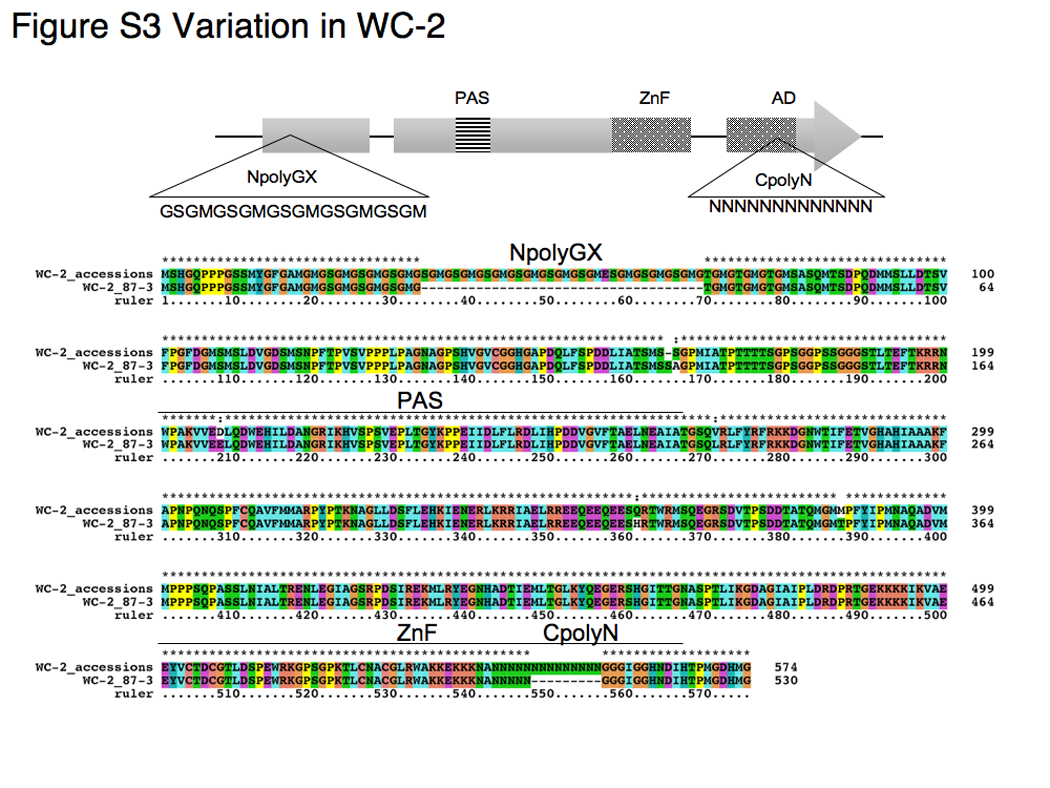

Supplement: Figure S3 — Variation in WC-2. Gene model for wc-2 including its domain coding structure: PAS, PAS domain; ZnF, zinc finger; and AD, activation domain. wc-2 has two SSR, the NpolyGX and CpolyN. WC-2 protein sequence from the sequence of two N. crassa accessions FGSC#3974 and 4832 and comparison to the 87-3 reference strain. Lines over protein sequence demarcate respective domains. Choice of accessions, sequencing and alignment in Clustalx are described in Experimental procedures. (2.50 MB TIF) [file pone.0000795.s003.tif]

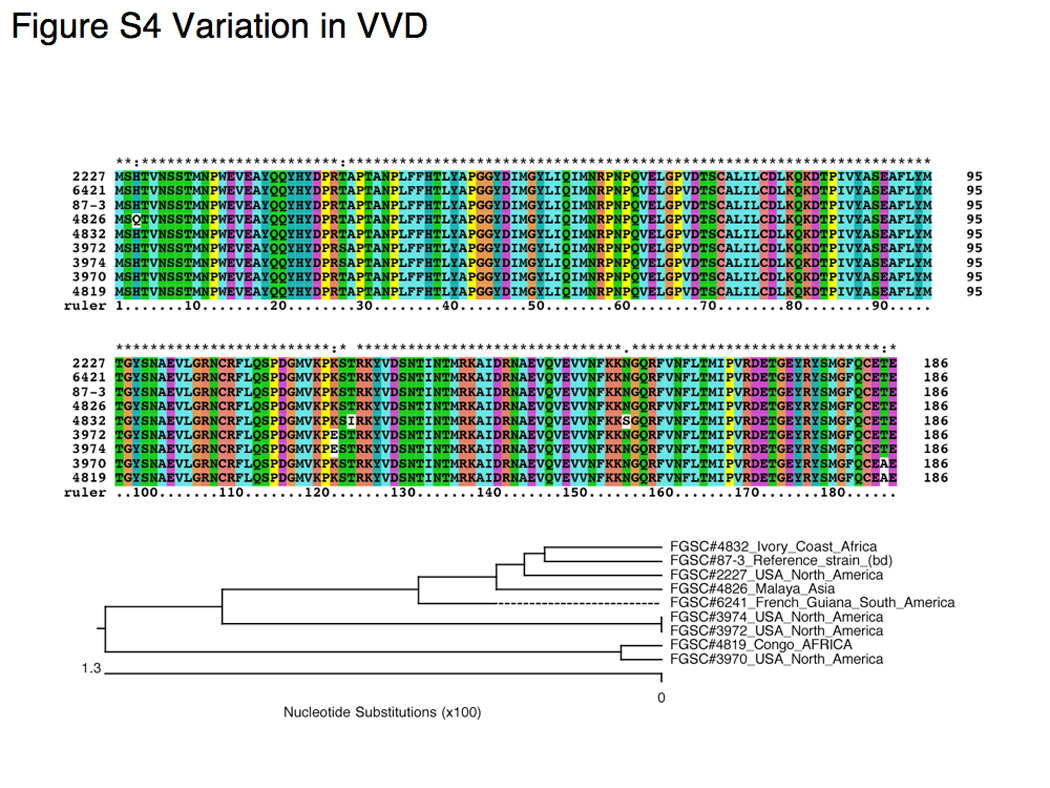

Supplement: Figure S4 — Variation in VVD. VVD protein sequence deduced from the sequence of N. crassa accessions FGSC#2227, 6421, 87-3 (a reference strain), 4826, 4832, 3972, 3974, 3970, and 4819. Phylogenetic tree of VVD proteins across accessions was created using ClustalW alignment in MegAlign DNASTAR Lasergene 6 software suite with default settings. Choice of accessions, sequencing and alignment in Clustalx are described in Experimental procedures. (2.50 MB TIF) [file pone.0000795.s004.tif]

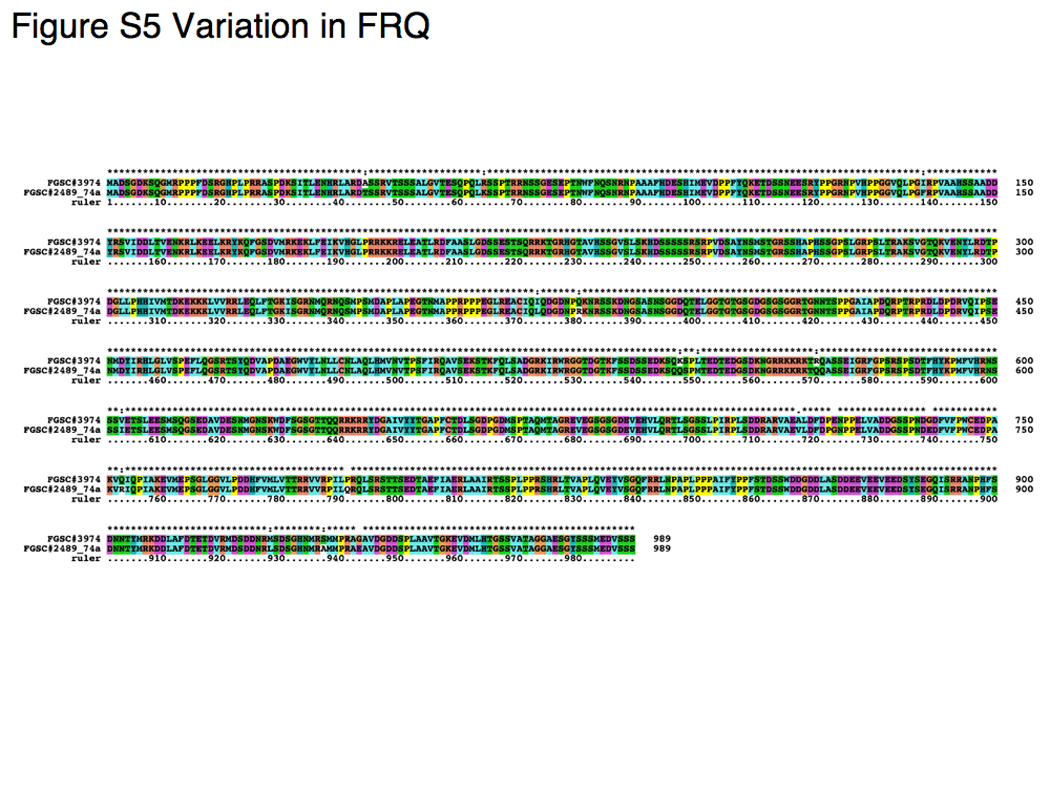

Supplement: Figure S5 — Variation in FRQ. FRQ protein sequence deduced from sequencing N. crassa accessions FGSC#3974 and sequenced lab strain 74a (FGSC#2489). Choice of accessions, sequencing and alignment in Clustalx are described in Experimental procedures. (2.50 MB TIF) [file pone.0000795.s005.tif]
